# Remote Monitoring of Patients with Retinal Vein Occlusions Treated with Anti-VEGF: A Pilot Study

**DOI:** 10.3390/jcm14072330

**Published:** 2025-03-28

**Authors:** Niccolò Castellino, Francesco Cappellani, Edoardo Dammino, Giovanni Rubegni, Davide Scollo, Andrea Russo, Teresio Avitabile, Antonio Longo

**Affiliations:** 1 Department of Ophthalmology, University of Catania, 95123 Catania, Italy; 2 Ophthalmology Unit, Department of Medicine, Surgery and Neurosciences, University of Siena, 51300 Siena, Italy

**Keywords:** retinal vein occlusion, anti-VEGF, telemedicine, optical coherence tomography, macular edema

## Abstract

**Purpose**: To assess the feasibility and effectiveness of remote monitoring for patients with retinal vein occlusion (RVO) treated with intravitreal anti-VEGF injections. **Methods**: A retrospective analysis was conducted at the Eye Clinic of the University of Catania. Thirty-four eyes of 34 patients with RVO were included for a 12-month follow-up period. After a comprehensive baseline ophthalmic examination, the patients received a loading treatment consisting of three monthly intravitreal injections of anti-VEGF, followed by monthly or bimonthly remote follow-up visits at peripheral centers. Optical coherence tomography (OCT) images and clinical data were shared online with our eye clinic for remote evaluations. Data on hospital and peripheral center visits, intravitreal injections, and OCT scans were collected and analyzed. **Results**: The patients had an average of 5.71 ± 1.14 visits to peripheral centers and 2.1 ± 0.8 visits to our center for fluorescein angiography. The mean number of injections was 5.26 ± 1.29 and the mean improvement in best-corrected visual acuity (BCVA) was 11.47 ± 5.56 letters. Remote OCT evaluations accounted for 194 scans, there was a high agreement between two expert in-hospital examinators (Cohen’s κ = 0.927) with only 14 cases requiring hospital visits for inconclusive results. **Conclusions**: Remote monitoring for RVO patients significantly reduced hospital admissions for follow-up visits, reducing the clinical burden on medical staff, patients, and caregivers, while maintaining reliable patient assessments.

## 1. Introduction

Retinal vein occlusions (RVOs) are a group of vascular diseases that represent the second most common retinal vascular disorder. There are two main types according to the involved outflow vessel: branch retinal vein occlusion (BRVO) and central retinal vein occlusion (CRVO) [1,2].

The incidence of RVO has been reported to vary with age, ethnicity, and underlying systemic conditions such as hypertension, diabetes, and cardiovascular disease, with the global prevalence estimated to be approximately 16 million cases [3]. In addition to systemic risk factors, glaucoma has been identified as a significant ocular risk factor for RVO [4]. CRVO typically has a more severe clinical course compared to BRVO, often leading to significant visual impairment if left untreated [5].

RVO is not just a localized ocular issue but reflects systemic vascular health, underscoring the need for a multidisciplinary approach in its management involving collaboration among ophthalmologists, internists, and cardiologists. Its clinical significance is compounded by the fact that the condition often coexists with other vascular diseases, such as stroke and myocardial infarction, which further highlight the importance of early diagnosis and comprehensive management [6].

One of the key pathological mechanisms in RVO is the upregulation of the Vascular Endothelial Growth Factor (VEGF) in response to retinal hypoxia caused by venous occlusion. VEGF dysregulation may lead to ischemic RVO, and the need for a patient to undergo fluorescein angiography (FA) examinations even over the course of follow-ups to promptly identify ischemic areas and treat them with laser photocoagulation [7,8]. VEGF plays a pivotal role in the pathogenesis of macular edema by increasing retinal vascular permeability. Anti-VEGF drugs administered by intravitreal injections have shown efficacy and safety in both BRVO and CRVO. In particular, the development of anti-VEGF agents has revolutionized the management of macular edema secondary to RVO, reducing the risk of vision loss and improving long-term outcomes [9,10,11,12]. Despite these advancements, the frequent need for monitoring and intravitreal injections poses a significant challenge for both patients and healthcare systems, particularly in resource-constrained settings where access to specialized care may be limited. Accordingly, the patients affected by RVO represent a significant treatment burden for eye units and require eye visits and optical coherence tomography (OCT) examinations in most cases on a monthly or bimonthly basis. The assiduity of the follow-up schedule places a considerable strain on healthcare systems, particularly in tertiary care centers, and affects patients’ quality of life and psychological well-being. Repeated injections and frequent hospital visits are often associated with anxiety, discomfort, and a sense of dependency, particularly among elderly individuals who are the most affected demographic. This can be physically and emotionally taxing for patients and their caregivers. Additionally, transportation barriers, financial constraints, and the time commitment required for regular hospital visits can lead to treatment fatigue, potentially affecting compliance and long-term outcomes [13]. Moreover, for patients with concurrent glaucoma, the burden of care is even greater, as they often require regular follow-ups with both retinal and glaucoma specialists for intraocular pressure monitoring and potential adjustments in glaucoma therapy.

The COVID pandemic forced us to promptly change the follow-up setting of patients affected by chronic disease by reducing the number of patient admissions to hospitals [14,15,16]. An unexpected, forced change often risks causing a shock both for patients and healthcare professionals. On the other hand, it may represent a tipping point to start planning a new follow-up modality for chronic patients without requiring a face-to-face assessment for every visit. During the pandemic, telemedicine emerged as a critical tool to maintain continuity of care while minimizing the risk of viral transmission. Its implementation in various fields of medicine demonstrated that remote monitoring could be a feasible, efficient, and scalable solution for managing chronic diseases. This experience highlighted the potential of telemedicine to address long-standing challenges in the management of chronic diseases like RVO, especially for patients requiring frequent monitoring and intervention.

In the last few years, telemedicine has demonstrated a valid approach to balance the accuracy of patient assessments with validated protocols and the need to limit patient admissions to tertiary referral centers [17,18,19,20]. In ophthalmology, advancements in imaging technology and digital platforms have expanded the scope of telemedicine, enabling detailed assessments of retinal pathology through remotely shared OCT scans. This innovation not only enhances diagnostic accuracy but also facilitates timely interventions. Accordingly, the expanding spectrum of telemedicine in ophthalmology represents a significant opportunity to reshape the follow-up modality of patients who underwent intravitreal injections.

Finally, this patient population may benefit from the online communication of retinal imaging examinations and clinical data, avoiding a significant number of hospital admissions. Furthermore, integrating telemedicine into routine care may improve patient compliance by offering more convenient follow-up options and reducing logistical challenges, such as travel and waiting times.

In the present study, we have assessed the feasibility of this clinical setting to better understand its limitations and potential in patients affected by RVO who underwent an intravitreal injection for macular edema.

## 2. Materials and Methods

A retrospective, clinical analysis evaluating the feasibility of a remote follow-up assessment for patients affected by RVO who underwent intravitreal injections (anti-VEGF) and other invasive procedures (laser therapy and fluorescein angiography).

All patients underwent a complete baseline ophthalmic examination at the Eye Clinic of the University of Catania, including a best-corrected visual acuity measurement, an anterior segment biomicroscopic examination, a fundus examination, OCT, and FA. All patients received a loading treatment consisting of three monthly intravitreal injections of anti-VEGF drugs. Subsequently, the patients received a series of monthly or bimonthly follow-up visits from an ophthalmologist at a peripheral eye center near their home. After completing the loading dose, all patients were referred to a peripheral center for follow-up monitoring. Four peripheral centers participated in this study. To maintain consistency in imaging acquisition and analysis, only the centers equipped with an Optovue OCT system (Optovue Inc., Fremont, CA, USA) were included. This ensured uniformity in scan quality, facilitating standardized remote assessments by the expert graders at our clinic. All intravitreal injections were performed exclusively in the operating room of our center. Patients who showed signs of persistent macular edema (intraretinal/subretinal fluid) or disease reactivation during the remote OCT assessments were recalled to our center for intravitreal injections. The process includes a vision acuity test, an intraocular pressure measurement, a biomicroscopic examination through a slit lamp, a fundus examination, and optical coherence tomography. All the relevant clinical data of the visit as well as the OCT images were shared via encrypted emails using a password to ensure data confidentiality with the macula service of the Eye Clinic of the University of Catania for a detailed re-examination. The OCT images were assessed by two expert eye specialists (N.C. and A.L.) to establish the need for additional intravitreal injections over the 12-month follow-up period according to the intraretinal and subretinal fluid detected by OCT with a pro-re-nata (PRN) treatment protocol. An interobserver agreement between the two graders was assessed using Cohen’s Kappa coefficient. The inclusion criteria were as follows: a diagnosis of central or branch retinal vein occlusion associated with macular edema in patients who underwent intravitreal anti-VEGF treatment with aflibercept; patients who consistently followed up at a peripheral center, with at least bimonthly OCT assessments performed; and patients who had access to high-quality imaging suitable for a remote evaluation. The exclusion criteria were as follows: patients who were switched to other drugs during the study period (ranibizumab, corticosteroids), patients in circumstances complicated by proliferative retinopathy due to RVO, patients affected by any other retinal diseases, and those with bad-quality imaging.

Optical coherence tomography was carried out by Optovue XR Avanti (Optovue Inc., Fremont, CA, USA) with an A-scan rate of 70,000 scans per second, a bandwidth of 50 nm, and a light source of 840 nm. All the scans were centered on the foveola, and a volumetric acquisition was performed by a retina map function. The disease activity observed by OCT was defined as the detection of subretinal fluid (the areas of low reflectivity in the subretinal space), intraretinal cysts (the presence of intraretinal areas of low reflectivity), and an increased Central Retinal Thickness (CRT). The latter was defined as the average macular thickness between its inner and outer borders on all A-scans taken in the central 1 mm sector. Based on the CRT measurements, the examiners at the hospital evaluated the progression and the treatment response in each patient who visited in a peripheral center to plan customized treatment schedules; in addition, qualitative retinal biomarkers were evaluated. If significant progression was detected, the patients were referred back to the hospital for further assessment and potential treatment adjustments. All patients were referred to a peripheral center for follow-up monitoring. The patients who showed signs of persistent macular edema (intraretinal/subretinal fluid) or disease reactivation during the remote OCT assessments were recalled to our center for intravitreal injections. Following treatment, these patients resumed follow-ups at their respective peripheral centers, where OCT monitoring continued.

The following data were collected in the analysis: visits to the hospital, visits to a peripheral center, intravitreal anti-VEGF injections, fluorescein angiographies, and laser treatments. FA was performed in all CRVO cases at baseline, at 3 months, and in cases where clinical need was identified, based on changes in OCT biomarkers or in visual acuity. In BRVO cases, FA was conducted at baseline. The patients requiring invasive procedures such as FA, laser photocoagulation, or intravitreal injections were referred back to our center. The OCT images were analyzed to assess the rate of further investigation at the hospital. All the collected data were merged using a standardized Excel version 2502 spreadsheet (Microsoft Corporation; Redmond, WA, USA). Agreement between observers in detecting disease activity via OCT as well as the need for further investigation was tested by Cohen’s kappa.

## 3. Results

Overall, 34 eyes of 34 patients were included in this study, with all patients completing the 12-month retrospective follow-up period. The patient population had a mean age of 67.56 ± 8.04 years, with 14 females (41.2%) and 20 males (58.8%). The baseline demographic and clinical characteristics are detailed in Table 1.

**Figure 1:**
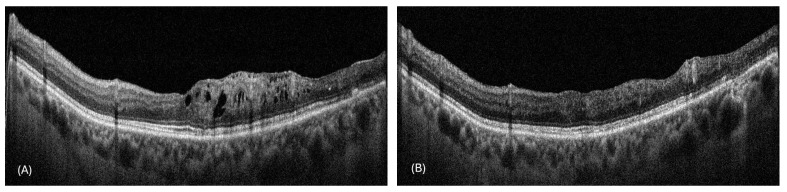
Representative horizontal OCT scans through the fovea of a patient with branch retinal vein occlusion captured at (**A**) baseline and (**B**) after a 3-month follow-up visit post intravitreal anti-VEGF treatment, after which OCT monitoring continued at the peripheral center.

**Figure 2:**
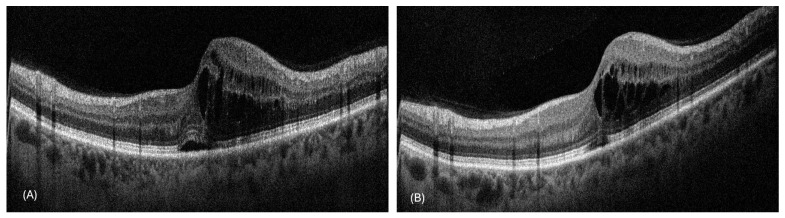
Representative horizontal OCT scans through the fovea of a patient with central retinal vein occlusion who followed up at a peripheral center, showing (**A**) baseline and (**B**) 3 months post-treatment. Persistent macular edema was observed, necessitating the patient’s return to our center for further intravitreal injections.

During the follow-ups, the patients had an average gain in BCVA of 11.47 ± 5.56 letters. The mean number of visits to the peripheral center per patient was 5.71 ± 1.14, while the mean number of visits to our center for fluorescein angiography was 2.1 ± 0.8. Remote OCT monitoring was performed for 194 scans, with 14 scans (7.2%) requiring reassessment at our center due to inconclusive findings (Figure 1 and Figure 2 provide representative examples of the OCT scans acquired during this study). Cohen’s kappa (κ) for interobserver agreement in detecting disease activity on an OCT image was 0.927 (*p* < 0.001).

Twelve patients (35.3%) underwent laser treatment during the follow-up period (10 patients with CRVO and 2 patients with BRVO), and the mean number of anti-VEGF injections administered was 5.26 ± 1.29 per patient. Additionally, the patients required a mean of 2.4 ± 1.2 visits to the hospital for consultations with other specialists, such as internists or cardiologists. Detailed follow-up and treatment outcomes are reported in Table 2.

## 4. Discussion

In this study, we found that the remote management of patients with RVO was successful in limiting hospital admissions over the follow-up period. This approach aligns with the global push toward patient-centered care, where the emphasis is on improving access to high-quality care while reducing unnecessary strains on healthcare resources. Reducing hospital admissions for follow-up visits allowed the patients to be referred primarily for invasive procedures, including fluorescein angiography, laser treatment, and intravitreal injections. Telemedicine has been employed in ophthalmology for many years; however, its primary application has been as a screening strategy for chronic diseases with significant epidemiological impact, such as Diabetic Retinopathy (DR) and Glaucoma [21,22,23]. In England, 2.7 million individuals are screened for DR, and an analysis by Leal et al. suggests the clinical relevance of OCT as an examination included in the diabetic screening program. The authors view OCT as an effective digital surveillance tool for detecting macular changes and complications, including cystoid macular edema [24]. The main reasons for OCT’s efficacy include its ability to analyze the retina layer by layer in a non-invasive manner, its high reproducibility with the same scan protocol, and its high sensitivity and specificity in detecting macular edema. Therefore, these benefits also apply to the assessment of patients with retinal vein occlusion. This makes OCT a cornerstone technology not only for diagnosis but also for monitoring disease progression and treatment efficacy.

The importance of regular OCT monitoring in optimizing outcomes for patients with retinal vein occlusion has been highlighted in the ALBATROS study. Schuster et al. (2023) demonstrated that patients undergoing frequent OCT examinations experienced better gains in best-corrected visual acuity, particularly in cases of branch retinal vein occlusion [25].

Telemedicine can facilitate a more efficient monitoring of disease progression by enabling remote OCT evaluations, reducing the burden of frequent in-person visits, and addressing logistical challenges, particularly for elderly patients [26]. In a study by Starr et al. (2019), which examined 83 eyes from 59 patients with exudative age-related macular degeneration (AMD), the authors demonstrated that telemedicine could effectively facilitate the management of patients receiving intravitreal anti-VEGF therapy. By integrating care between retinal specialists and local ophthalmologists, they reduced the need for frequent long-distance travel while maintaining effective disease monitoring and treatment outcomes. These findings align with the potential benefits of telemedicine in managing retinal vein occlusion, particularly in reducing the logistical and emotional burdens associated with frequent follow-ups and treatment sessions [27].

In our analysis, we found a very limited number of cases (7.2%) where an OCT examination performed outside the hospital macula clinic was not adequately informative. In those cases, the patients were reassessed in the hospital clinic for further investigation. Additionally, we found a high agreement between the investigators (Cohen’s κ = 0.927) suggesting a validated scan protocol with simple imaging biomarkers and parameters, making the remote reassessment of OCT images extremely reproducible.

Despite these promising results, it is important to address the limitations of telemedicine in managing RVO. For instance, retinal ischemia, which often accompanies CRVO, cannot be adequately assessed without fluorescein angiography. Furthermore, patients with extensive ischemia require close monitoring and may not be suitable candidates for remote follow-ups. Developing clear criteria to stratify patients based on disease severity and risk factors is essential to optimize the use of telemedicine in this population. In addition, there is a need to ensure adequate training for clinicians at peripheral centers to maintain the quality and reliability of remote monitoring systems.

Retinal vein occlusion is a retinal vasculopathy that represents one of the main causes of anti-VEGF intravitreal injection administration. Randomized controlled trials have shown the efficacy and safety of these drugs in both the subtypes (BRVO and CRVO) [28,29,30,31,32,33]. However, many injections are often required to stabilize the clinical picture. Because of the assiduity of treatments and visits, RVO is a macular disease that can significantly benefit from remote monitoring for non-invasive procedures. Furthermore, the RVO population often consists of elderly individuals with cardiovascular comorbidities [34,35,36]. According to our results, they had an average of 2.4 hospital admissions a year for follow-up visits and/or examinations related to cardiovascular conditions. This demographic is particularly vulnerable to complications from frequent hospital visits due to their higher susceptibility to infections, limited mobility, and the potential exacerbation of existing health conditions caused by the stress of travel and prolonged waiting times. Minimizing hospital visits not only reduces these risks but also enhances their quality of life by providing more accessible and patient-friendly care options. For instance, frequent hospital trips may increase the likelihood of adverse events such as falls, hospital-acquired infections, and the exacerbation of cardiovascular symptoms. By implementing remote monitoring, patients can receive personalized care in a safer and more controlled environment, reducing these potential complications. For this reason, reducing additional hospital visits is of pivotal importance for these patients, who already have an extraocular significant treatment burden.

However, RVO is a complex disease where macular edema and retinal ischemia may occur suddenly, and it requires a balance between digital surveillance and hospital examinations to perform fluorescein angiography, especially in patients affected by CRVO, which is more frequently associated with retinal ischemia. It is important to consider that RVO represents one-third of the causes of neovascular glaucoma, which is the final stage of an untreated retinal ischemic process that leads to inflammation and complete visual loss [37,38]. In our analysis, the patients visited the hospital, on average, two times per year for fluorescein angiography. We focused digital surveillance on the patients affected by RVO complicated by macular edema. The patients affected by extensive ischemia complicated by proliferative retinopathy are not suitable candidates for follow-ups through this clinical setting and should be followed up with only at a macula clinic. The present study is, to the best of our knowledge, the first report of remote monitoring in patients affected by RVO who underwent intravitreal anti-VEGF injections.

In our study, we observed a mean BCVA improvement of 11.5 letters after 12 months of follow-up, with a mean number of injections of 5.3 ± 1.3. Randomized clinical trials have investigated the efficacy of intravitreal aflibercept in patients with retinal vein occlusion using conventional in-person follow-ups. In the VIBRANT trial, a phase 3 randomized clinical trial evaluating aflibercept in patients with macular edema following BRVO, the mean BCVA improvement at 12 months was 17.1 letters, with a mean of 9.0 ± 1.8 injections administered over the course of the first year [39]. Similarly, the COPERNICUS trial, a randomized phase 3 study involving patients with macular edema following CRVO, reported a mean BCVA gain of 16.2 letters at 1 year, with patients receiving 6 fixed monthly intravitreal aflibercept injections during the first 24 weeks, followed by a mean of 2.7 ± 1.7 injections [32]. In a real-world clinical setting, Chatziralli et al. reported a mean BCVA improvement of 8.3 letters after 12 months in patients with RVO-related macular edema treated with aflibercept [40].

We acknowledge that this is a pilot study and has several limitations, including the retrospective analysis, the absence of a comparison of the outcomes with a control group followed exclusively at the hospital, and that the patients were assessed outside the hospital clinic in different eye centers. Moreover, FA was required for the assessment of ischemic areas, necessitating hospital visits for certain patients. However, OCTA has emerged as a promising non-invasive imaging technique for evaluating retinal vascular changes without the need for dye-based angiography [41]. Although OCTA does not yet fully replace FA in detecting peripheral ischemia, its integration into remote monitoring protocols may help further reduce hospital visits in the future. Future studies should explore the feasibility of incorporating OCTA image transmission for remote vascular assessment, which could further optimize patient management and minimize the need for in-person fluorescein angiography.

In conclusion, RVOs have been shown to be retinal conditions well suited for remote monitoring, especially for patients affected by macular edema who undergo intravitreal injections.

## Figures and Tables

**Table 1:** Baseline demographic and clinical characteristics.

Number of Patients Who Completed Follow-Up (12 Months)	*n*	34
Mean age	years	67.56 ± 8.04
Females	*n* (%)	14 (41.2%)
Males	*n* (%)	20 (58.8%)
Number of eyes affected by CRVO	*n* (%)	10 (29.4%)
Number of eyes affected by BRVO	*n* (%)	24 (70.6%)
Patients who underwent cataract surgery during follow-up	*n* (%)	2 (5.9%)

CRVO = Central Retinal Vein Occlusion; BRVO = Branch Retinal Vein Occlusion.

**Table 2:** Twelve-month follow-up and treatment outcomes.

Mean gain in BCVA	letters	11.47 ± 5.56
Number of visits to a peripheral center per patient	mean	5.71 ± 1.14
Mean number of injections	mean	5.26 ± 1.29
Number of visits to our center for fluorescein angiography	mean	2.1 ± 0.8
Number of eyes that underwent laser treatment at our center	*n* (%)	12 (35.3%) 34 admissions
Number of OCT scans evaluated via remote monitoring	*n*	194
Number of visits to our center because OCT in the peripheral center was inconclusive	*n*	14 (7.2%)
Number of visits to our hospital for other specialist consultations (internist, cardiologist)	mean	2.4 ± 1.2

BCVA = Best-Corrected Visual Acuity; OCT = Optical Coherence Tomography.

## Data Availability

The data presented in this study are available on request from the authors.

## Abbreviations

The following abbreviations are used in this manuscript:

RVO	Retinal Vein Occlusion
BRVO	Branch Retinal Vein Occlusion
CRVO	Central Retinal Vein Occlusion
VEGF	Vascular Endothelial Growth Factor
FA	Fluorescein Angiography
OCT	Optical Coherence Tomography
BCVA	Best-Corrected Visual Acuity
CRT	Central Retinal Thickness
DR	Diabetic Retinopathy
PRN	Pro-Re-Nata
